# Tick-Borne Encephalitis in Sheep, Romania

**DOI:** 10.3201/eid2312.170166

**Published:** 2017-12

**Authors:** Jiri Salat, Andrei D. Mihalca, Marian Mihaiu, David Modrý, Daniel Ruzek

**Affiliations:** Veterinary Research Institute, Brno, Czech Republic (J. Salat, D. Ruzek);; University of Agricultural Sciences and Veterinary Medicine, Cluj-Napoca, Romania (A.D. Mihalca, M. Mihaiu);; Faculty of Veterinary Medicine and CEITEC VFU, University of Veterinary and Pharmaceutical Sciences, Brno (D. Modrý);; Institute of Parasitology, Biology Centre of the Czech Academy of Sciences, Ceske Budejovice, Czech Republic (D. Ruzek)

**Keywords:** Tick-borne encephalitis, tick-borne encephalitis virus, sheep, seroprevalence, zoonoses, sentinel, Romania, Transylvania, North-West Romania, viruses

## Abstract

Little is known about the occurrence of tick-borne encephalitis in Romania. Sheep are an infection source for humans and are useful sentinels for risk analysis. We demonstrate high antibody prevalence (15.02%) among sheep used as sentinels for this disease in 80% of the tested localities in 5 counties of northwestern Romania.

Tick-borne encephalitis (TBE) virus (family *Flaviviridae*, genus *Flavivirus*), is a zoonotic pathogen that causes severe neurologic disease in humans. In Europe, most cases of TBE are reported in Scandinavia and in countries in Central and Eastern Europe countries, but little is known about the current TBE epidemiologic situation in Romania.

The main tick vector for TBE virus, *Ixodes ricinus*, prefers leaf litter and the lower vegetation layers of temperate deciduous and mixed forests. In areas with high rainfall, *I. ricinus* ticks also occur in high densities in coniferous forests and in open areas such as grasslands (*1*). *I. ricinus* ticks are widely distributed in Romania (*2*) and are the most common ticks found on a variety of vertebrate hosts, including humans (*3**,**4*). 

Large domestic animals such as goats, sheep, and cattle are potential hosts for *I. ricinus* ticks. These animals may also develop an antibody response after infection with TBE virus without showing clinical signs and thus are a source of TBE virus among humans who consume nonpasteurized milk and milk products, which makes them valuable sentinels for the identification of TBE risk areas (*5*). 

Romania has the third largest sheep flock in the European Union (EU), after Great Britain and Spain, totaling ≈9.5 million sheep, which accounts for 11% of the total EU flock. Most of the animals are used for milk and meat production. The purpose of this study was to determine the current TBE virus infection status in northwestern Romania by using sheep as sentinels for TBE virus circulation.

## The Study

In September 2016, we randomly selected 519 serum aliquots from adult sheep from samples previously collected (July–August 2016) by the National Program for Surveillance, Prevention, Control and Eradication of Animal Diseases in 5 counties in northwestern Romania (Figure). The counties, number of samples, and number of sampling sites were Bihor, 119 samples, 12 locations; Bistrița-Năsăud, 100 samples, 10 locations; Cluj, 100 samples, 7 locations; Mureș, 100 samples, 11 locations; and Sălaj, 100 samples, 10 locations). We froze the aliquots at −20°C before processing. We used the IMMUNOZYM FSME (TBE) IgG All-Species kit (Progen GmbH, Heidelberg, Germany) ELISA to detect TBE virus antibodies. We retested samples exhibiting ≥25 Vienna units/mL by using virus neutralization test (VNT) as described previously (*6*).

**Figure:**
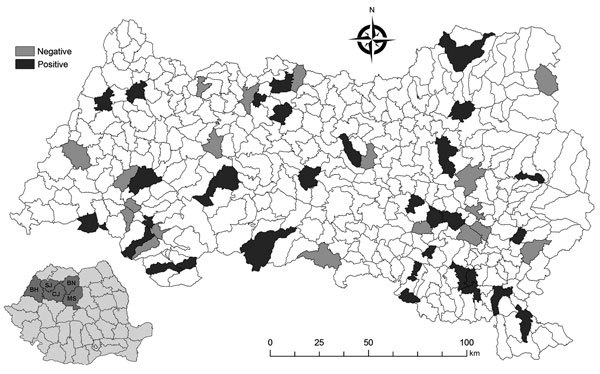
Locations in northwestern Romania where sheep were positive for tick-borne encephalitis virus–specific antibodies, determined by using virus neutralization test (*6*). BH, Bihor; BN, Bistriţa-Năsăud; CJ, Cluj; MS, Mureş; SJ, Sălaj.

We tested samples from 168 (32.37%) sheep with borderline or positive results from ELISA by using VNT, and this method identified 78 (15.02%) animals as positive for antibodies specifically neutralizing TBE virus (Technical Appendix Table). Positive results were distributed among the counties we investigated: the highest seroprevalence was identified in sheep in Bihor County (27.73%) and the lowest seroprevalence in Mureș County (2%) (Table; Figure). We found positive sheep samples in 40 (80%) of 50 examined locations.

**Table:** Seroprevalence of tick-borne encephalitis in sheep, northwestern Romania*

County	No. samples	No. locations	ELISA-positive samples, %†	VNT-positive samples, %	No. VNT-positive locations
Bihor	119	12	29.41	27.73	11
Bistrița-Năsăud	100	10	18.00	12.00	5
Cluj	100	7	67.00	11.00	5
Mureș	100	11	16.00	2.00	2
Sălaj	100	10	32.00	20.00	9
Total	519	50	32.37	15.2	32

*Testing locations were within communes subdivision of counties in Romania. †Includes all positive and suggestive samples with antibody titer ≥25 Vienna units /mL.

## Conclusions

TBE has been reported previously in Romania, but few studies confirmed the presence of the virus (*7**–**10*). However, in the past 3 decades, except for a few occasional records (*11*) and small-scale serologic surveys during clinical outbreaks, there have been large knowledge gaps in the studies on TBE in Romania, and the prevalence and distribution remain fairly unknown. We used sheep as sentinels for TBE virus distribution in northwestern Romania and identified the virus by VNT in 15.02% of sheep in 40 of 50 examined localities in 5 counties.

According to the latest technical report by the European Centre for Disease Control and Prevention (*12*), during 2008–2010, only 14 cases of TBE were reported among humans in Romania. All 14 cases originated in 5 counties in northwestern Romania; incidence was low (<0.5/100,000 inhabitants) in Bihor, Bistrița-Năsăud, Cluj, and Mureș and moderate (0.5–4.0/100,000 inhabitants) in Sălaj. In a recent survey done by collecting *I. ricinus* ticks from vegetation, livestock, and reptiles in central and southeastern Romania, the prevalence the TBE virus estimated by using molecular methods was <1% (*13*). Coipan et al. evaluated the seropositivity for TBE in 51 patients who had neurologic signs and were admitted to a hospital in Sibiu County, Romania; specific TBE antibodies were found in samples from 38 (75%) patients (*14*). An outbreak of rural TBE was reported in the central Romania region of Transylvania, related to consumption of infected goat milk; specific serology in 41.5% of the patients was positive for TBE virus (*15*). Similar seroprevalence studies in other outbreaks found lower seropositivity values (5.8%–11.6%) in human patients (*15*); the same study randomly evaluated 1,669 human serum samples from Transylvania and reported a 0.5% seroprevalence for TBE. Taken together, these results suggest that clinical cases of TBE in Romania are largely underreported, and many case-patients are misdiagnosed.

Our study confirms that TBE virus is endemic in northwestern Romania and should be considered a public health risk in this country. Considering the number of sheep and their almost ubiquitous presence in this country, their sentinel role in countrywide mapping of the TBE distribution should be evaluated.

Technical Appendix Results from the seroprevalence study of tick-borne encephalitis virus in sheep in northwestern Romania.
