# Supplementary material for: Tick-Borne Encephalitis in Sheep, Romania
Source: Emerg Infect Dis. 2017 Dec;23(12):2065–7. doi: 10.3201/eid2312.170166 (PMC5708217; doi:10.3201/eid2312.170166)
Supplement: Technical Appendix — Results from the seroprevalence study of tick-borne encephalitis virus in sheep in northwestern Romania. [file 17-0166-Techapp-s1.pdf]

Publisher: CDC; Journal: Emerging Infectious Diseases
Article Type: Dispatch; Volume: 23; Issue: 12; Year: 2017; Article ID: 17-0166
DOI: 10.3201/eid2312.170166; TOC Head: Dispatch
Article DOI: https://doi.org/10.3201/eid2312.170166

Tick-Borne Encephalitis in Sheep, Romania

Technical Appendix

Technical Appendix Table. Results from the seroprevalence study of tick-borne encephalitis in sheep in northwestern Romania.

Sample no.	County	County abbreviation	Commune	Locality	ELISA (VIEU/ml)	ELISA result	VNT titer	VNT result
1	Bihor	BH	Marghita	Marghita	24.27	–	NA	NA
2	Bihor	BH	Marghita	Marghita	22.11	–	NA	NA
3	Bihor	BH	Marghita	Marghita	20.22	–	NA	NA
4	Bihor	BH	Marghita	Marghita	21.03	–	NA	NA
5	Bihor	BH	Marghita	Marghita	28.86	+	40	+
6	Bihor	BH	Marghita	Marghita	20.22	–	NA	NA
7	Bihor	BH	Marghita	Marghita	23.73	–	NA	NA
8	Bihor	BH	Marghita	Marghita	22.38	–	NA	NA
9	Bihor	BH	Marghita	Marghita	29.41	+	20	+
10	Bihor	BH	Marghita	Marghita	22.11	–	NA	NA
11	Bihor	BH	Diosig	Diosig	31.03	+	40	+
12	Bihor	BH	Diosig	Diosig	25.62	+	20	+
13	Bihor	BH	Diosig	Diosig	19.41	–	NA	NA
14	Bihor	BH	Diosig	Diosig	19.41	–	NA	NA
15	Bihor	BH	Diosig	Diosig	22.92	–	NA	NA
16	Bihor	BH	Diosig	Diosig	25.08	+	80	+
17	Bihor	BH	Diosig	Diosig	24.00	–	NA	NA
18	Bihor	BH	Diosig	Diosig	23.46	–	NA	NA
19	Bihor	BH	Diosig	Diosig	28.05	+	20	+
20	Bihor	BH	Diosig	Diosig	16.16	–	NA	NA
21	Bihor	BH	Salonta	Salonta	14.54	–	NA	NA
22	Bihor	BH	Salonta	Salonta	15.62	–	NA	NA
23	Bihor	BH	Salonta	Salonta	14.54	–	NA	NA
24	Bihor	BH	Salonta	Salonta	14.81	–	NA	NA
25	Bihor	BH	Salonta	Salonta	18.05	–	NA	NA
26	Bihor	BH	Salonta	Salonta	16.97	–	NA	NA
27	Bihor	BH	Salonta	Salonta	18.05	–	NA	NA
28	Bihor	BH	Salonta	Salonta	17.51	–	NA	NA
29	Bihor	BH	Salonta	Salonta	23.19	–	NA	NA
30	Bihor	BH	Salonta	Salonta	15.62	–	NA	NA
31	Bihor	BH	Nojorid	Nojorid	18.86	–	NA	NA
32	Bihor	BH	Nojorid	Nojorid	26.97	+	20	+
33	Bihor	BH	Nojorid	Nojorid	25.62	+	40	+
34	Bihor	BH	Nojorid	Nojorid	20.49	–	NA	NA
35	Bihor	BH	Nojorid	Nojorid	26.43	+	40	+
36	Bihor	BH	Nojorid	Nojorid	39.41	+	20	+
37	Bihor	BH	Nojorid	Nojorid	33.46	+	80	+
38	Bihor	BH	Nojorid	Nojorid	17.51	–	NA	NA
39	Bihor	BH	Nojorid	Nojorid	24.54	–	NA	NA
40	Bihor	BH	Nojorid	Nojorid	20.22	–	NA	NA
41	Bihor	BH	Lugasu de Jos	Lugasu de Jos	19.41	–	NA	NA
42	Bihor	BH	Lugasu de Jos	Lugasu de Jos	26.97	+	80	+
43	Bihor	BH	Lugasu de Jos	Lugasu de Jos	17.24	–	NA	NA
44	Bihor	BH	Lugasu de Jos	Lugasu de Jos	16.43	–	NA	NA
45	Bihor	BH	Lugasu de Jos	Lugasu de Jos	16.97	–	NA	NA
46	Bihor	BH	Lugasu de Jos	Lugasu de Jos	16.43	–	NA	NA
47	Bihor	BH	Lugasu de Jos	Lugasu de Jos	18.59	–	NA	NA
48	Bihor	BH	Lugasu de Jos	Lugasu de Jos	38.05	+	20	+
49	Bihor	BH	Lugasu de Jos	Lugasu de Jos	21.57	–	NA	NA
50	Bihor	BH	Lugasu de Jos	Lugasu de Jos	16.43	–	NA	NA

Sample no.	County	County abbreviation	Commune	Locality	ELISA (VIEU/ml)	ELISA result	VNT titer	VNT result
51	Bihor	BH	Rosiori	Rosiori	22.65	–	NA	NA
52	Bihor	BH	Rosiori	Rosiori	24.00	–	NA	NA
53	Bihor	BH	Rosiori	Rosiori	16.43	–	NA	NA
54	Bihor	BH	Rosiori	Rosiori	21.03	–	NA	NA
55	Bihor	BH	Rosiori	Rosiori	15.35	–	NA	NA
56	Bihor	BH	Rosiori	Rosiori	28.32	+	80	+
57	Bihor	BH	Rosiori	Rosiori	17.78	–	NA	NA
58	Bihor	BH	Rosiori	Rosiori	25.89	+	40	+
59	Bihor	BH	Rosiori	Rosiori	19.14	–	NA	NA
60	Bihor	BH	Rosiori	Rosiori	19.95	–	NA	NA
61	Bihor	BH	Sanmartin	Sanmartin	14.27	–	NA	NA
62	Bihor	BH	Sanmartin	Sanmartin	15.62	–	NA	NA
63	Bihor	BH	Sanmartin	Sanmartin	13.46	–	NA	NA
64	Bihor	BH	Sanmartin	Sanmartin	35.89	+	20	+
65	Bihor	BH	Sanmartin	Sanmartin	17.24	–	NA	NA
66	Bihor	BH	Sanmartin	Sanmartin	14.00	–	NA	NA
67	Bihor	BH	Sanmartin	Sanmartin	18.05	–	NA	NA
68	Bihor	BH	Sanmartin	Sanmartin	18.32	–	NA	NA
69	Bihor	BH	Sanmartin	Sanmartin	17.78	–	NA	NA
70	Bihor	BH	Sanmartin	Sanmartin	14.54	–	NA	NA
71	Bihor	BH	Vascau	Vascau	28.59	+	40	+
72	Bihor	BH	Vascau	Vascau	27.78	+	20	+
73	Bihor	BH	Vascau	Vascau	26.16	+	40	+
74	Bihor	BH	Vascau	Vascau	24.81	–	NA	NA
75	Bihor	BH	Vascau	Vascau	18.05	–	NA	NA
76	Bihor	BH	Vascau	Vascau	26.16	+	10	–
77	Bihor	BH	Vascau	Vascau	19.68	–	NA	NA
78	Bihor	BH	Vascau	Vascau	58.32	+	80	+
79	Bihor	BH	Vascau	Vascau	84.81	+	AF	–
80	Bihor	BH	Vascau	Vascau	27.51	+	40	+
81	Bihor	BH	Simian	Simian	25.89	+	80	+
82	Bihor	BH	Simian	Simian	16.43	–	NA	NA
83	Bihor	BH	Simian	Simian	24.54	–	NA	NA
84	Bihor	BH	Simian	Simian	27.78	+	40	+
85	Bihor	BH	Simian	Simian	17.51	–	NA	NA
86	Bihor	BH	Simian	Simian	14.27	–	NA	NA
87	Bihor	BH	Simian	Simian	17.51	–	NA	NA
88	Bihor	BH	Simian	Simian	23.46	–	NA	NA
89	Bihor	BH	Simian	Simian	23.00	–	NA	NA
90	Bihor	BH	Simian	Simian	16.94	–	NA	NA
91	Bihor	BH	Cefa	Cefa	31.22	+	40	+
92	Bihor	BH	Cefa	Cefa	25.10	+	20	+
93	Bihor	BH	Cefa	Cefa	21.28	–	NA	NA
94	Bihor	BH	Cefa	Cefa	22.16	–	NA	NA
95	Bihor	BH	Cefa	Cefa	21.11	–	NA	NA
96	Bihor	BH	Cefa	Cefa	27.60	+	20	+
97	Bihor	BH	Cefa	Cefa	25.14	+	20	+
98	Bihor	BH	Cefa	Cefa	23.21	–	NA	NA
99	Bihor	BH	Cefa	Cefa	26.37	+	20	+
100	Bihor	BH	Cefa	Cefa	24.26	–	NA	NA
101	Bihor	BH	Tulca	Tulca	17.95	–	NA	NA
102	Bihor	BH	Tulca	Tulca	17.07	–	NA	NA
103	Bihor	BH	Tulca	Tulca	16.72	–	NA	NA
104	Bihor	BH	Tulca	Tulca	16.72	–	NA	NA
105	Bihor	BH	Tulca	Tulca	25.49	+	40	+
106	Bihor	BH	Tulca	Tulca	26.89	+	20	+
107	Bihor	BH	Tulca	Tulca	19.35	–	NA	NA
108	Bihor	BH	Tulca	Tulca	17.60	–	NA	NA
109	Bihor	BH	Tulca	Tulca	19.88	–	NA	NA
110	Bihor	BH	Tulca	Tulca	21.98	–	NA	NA
111	Bihor	BH	Tauteu	Tauteu	14.61	–	NA	NA
112	Bihor	BH	Tauteu	Tauteu	16.72	–	NA	NA
113	Bihor	BH	Tauteu	Tauteu	17.77	–	NA	NA
114	Bihor	BH	Tauteu	Tauteu	28.82	+	20	+
115	Bihor	BH	Tauteu	Tauteu	23.74	–	NA	NA
116	Bihor	BH	Tauteu	Tauteu	22.33	–	NA	NA
117	Bihor	BH	Tauteu	Tauteu	18.30	–	NA	NA

Sample no.	County	County abbreviation	Commune	Locality	ELISA (VIEU/ml)	ELISA result	VNT titer	VNT result
118	Bihor	BH	Tauteu	Tauteu	26.54	+	40	+
119	Bihor	BH	Tauteu	Tauteu	32.51	+	40	+
120	Bistrita Nasaud	BN	Zagra	Zagra	13.61	–	NA	NA
121	Bistrita Nasaud	BN	Zagra	Zagra	13.61	–	NA	NA
122	Bistrita Nasaud	BN	Zagra	Zagra	17.24	–	NA	NA
123	Bistrita Nasaud	BN	Zagra	Zagra	15.73	–	NA	NA
124	Bistrita Nasaud	BN	Zagra	Zagra	15.42	–	NA	NA
125	Bistrita Nasaud	BN	Zagra	Zagra	13.91	–	NA	NA
126	Bistrita Nasaud	BN	Zagra	Zagra	16.94	–	NA	NA
127	Bistrita Nasaud	BN	Zagra	Zagra	14.82	–	NA	NA
128	Bistrita Nasaud	BN	Zagra	Zagra	17.55	–	NA	NA
129	Bistrita Nasaud	BN	Zagra	Zagra	15.73	–	NA	NA
130	Bistrita Nasaud	BN	Uriu	Cristestii Ciceului	5.12	–	NA	NA
131	Bistrita Nasaud	BN	Uriu	Cristestii Ciceului	14.82	–	NA	NA
132	Bistrita Nasaud	BN	Uriu	Cristestii Ciceului	17.85	–	NA	NA
133	Bistrita Nasaud	BN	Uriu	Cristestii Ciceului	15.12	–	NA	NA
134	Bistrita Nasaud	BN	Uriu	Uriu	20.88	–	NA	NA
135	Bistrita Nasaud	BN	Uriu	Uriu	25.12	+	80	+
136	Bistrita Nasaud	BN	Uriu	Hasmasu Ciceului	36.33	+	40	+
137	Bistrita Nasaud	BN	Uriu	Hasmasu Ciceului	23.30	–	NA	NA
138	Bistrita Nasaud	BN	Uriu	Hasmasu Ciceului	20.27	–	NA	NA
139	Bistrita Nasaud	BN	Uriu	Ilisua	26.33	+	40	+
140	Bistrita Nasaud	BN	Chiuza	Sasarm	14.82	–	NA	NA
141	Bistrita Nasaud	BN	Chiuza	Sasarm	56.33	+	20	+
142	Bistrita Nasaud	BN	Chiuza	Sasarm	12.09	–	NA	NA
143	Bistrita Nasaud	BN	Chiuza	Sasarm	11.79	–	NA	NA
144	Bistrita Nasaud	BN	Chiuza	Sasarm	15.42	–	NA	NA
145	Bistrita Nasaud	BN	Chiuza	Sasarm	14.82	–	NA	NA
146	Bistrita Nasaud	BN	Chiuza	Sasarm	14.82	–	NA	NA
147	Bistrita Nasaud	BN	Chiuza	Sasarm	21.18	–	NA	NA
148	Bistrita Nasaud	BN	Chiuza	Sasarm	35.73	+	20	+
149	Bistrita Nasaud	BN	Chiuza	Sasarm	37.24	+	80	+
150	Bistrita Nasaud	BN	Salva	Salva	15.73	–	NA	NA
151	Bistrita Nasaud	BN	Salva	Salva	23.30	–	NA	NA
152	Bistrita Nasaud	BN	Salva	Salva	13.91	–	NA	NA
153	Bistrita Nasaud	BN	Salva	Salva	12.09	–	NA	NA
154	Bistrita Nasaud	BN	Salva	Salva	23.00	–	NA	NA
155	Bistrita Nasaud	BN	Salva	Salva	27.24	+	20	+
156	Bistrita Nasaud	BN	Salva	Salva	34.82	+	40	+
157	Bistrita Nasaud	BN	Salva	Salva	33.30	+	20	+
158	Bistrita Nasaud	BN	Salva	Salva	18.15	–	NA	NA
159	Bistrita Nasaud	BN	Salva	Salva	323.91	+	40	+
160	Bistrita Nasaud	BN	Sieu-Odorhei	Sirioara	15.12	–	NA	NA
161	Bistrita Nasaud	BN	Sieu-Odorhei	Sirioara	6.94	–	NA	NA
162	Bistrita Nasaud	BN	Sieu-Odorhei	Sirioara	16.64	–	NA	NA
163	Bistrita Nasaud	BN	Sieu-Odorhei	Sirioara	20.58	–	NA	NA
164	Bistrita Nasaud	BN	Sieu-Odorhei	Sirioara	14.52	–	NA	NA
165	Bistrita Nasaud	BN	Sieu-Odorhei	Sirioara	15.73	–	NA	NA
166	Bistrita Nasaud	BN	Sieu-Odorhei	Sirioara	17.85	–	NA	NA
167	Bistrita Nasaud	BN	Sieu-Odorhei	Sirioara	39.06	+	20	+
168	Bistrita Nasaud	BN	Sieu-Odorhei	Sirioara	48.15	+	10	–
169	Bistrita Nasaud	BN	Sieu-Odorhei	Sirioara	12.70	–	NA	NA
170	Bistrita Nasaud	BN	Chiochis	Strugureni	15.12	–	NA	NA
171	Bistrita Nasaud	BN	Chiochis	Strugureni	12.70	–	NA	NA
172	Bistrita Nasaud	BN	Chiochis	Chiochis	26.03	+	AF	–
173	Bistrita Nasaud	BN	Chiochis	Strugureni	16.33	–	NA	NA
174	Bistrita Nasaud	BN	Chiochis	Bozies	15.42	–	NA	NA
175	Bistrita Nasaud	BN	Chiochis	Chiochis	13.91	–	NA	NA
176	Bistrita Nasaud	BN	Chiochis	Bozies	37.85	+	AF	–
177	Bistrita Nasaud	BN	Chiochis	Chiochis	11.18	–	NA	NA
178	Bistrita Nasaud	BN	Chiochis	Apatiu	24.82	–	NA	NA
179	Bistrita Nasaud	BN	Chiochis	Apatiu	27.55	+	AF	–
180	Bistrita Nasaud	BN	Urmenis	Sopteriu	28.45	+	10	–
181	Bistrita Nasaud	BN	Urmenis	Sopteriu	22.70	–	NA	NA

Sample no.	County	County abbreviation	Commune	Locality	ELISA (VIEU/ml)	ELISA result	VNT titer	VNT result
182	Bistrita Nasaud	BN	Urmenis	Sopteriu	11.79	–	NA	NA
183	Bistrita Nasaud	BN	Urmenis	Sopteriu	20.58	–	NA	NA
184	Bistrita Nasaud	BN	Urmenis	Sopteriu	25.12	+	10	–
185	Bistrita Nasaud	BN	Urmenis	Sopteriu	8.45	–	NA	NA
186	Bistrita Nasaud	BN	Urmenis	Sopteriu	17.55	–	NA	NA
187	Bistrita Nasaud	BN	Urmenis	Sopteriu	18.15	–	NA	NA
188	Bistrita Nasaud	BN	Urmenis	Sopteriu	19.06	–	NA	NA
189	Bistrita Nasaud	BN	Urmenis	Sopteriu	14.52	–	NA	NA
190	Bistrita Nasaud	BN	Josenii Bargaului	Josenii Bargaului	18.76	–	NA	NA
191	Bistrita Nasaud	BN	Josenii Bargaului	Josenii Bargaului	12.70	–	NA	NA
192	Bistrita Nasaud	BN	Josenii Bargaului	Josenii Bargaului	20.27	–	NA	NA
193	Bistrita Nasaud	BN	Josenii Bargaului	Josenii Bargaului	13.00	–	NA	NA
194	Bistrita Nasaud	BN	Josenii Bargaului	Josenii Bargaului	13.00	–	NA	NA
195	Bistrita Nasaud	BN	Josenii Bargaului	Josenii Bargaului	13.00	–	NA	NA
196	Bistrita Nasaud	BN	Josenii Bargaului	Josenii Bargaului	13.00	–	NA	NA
197	Bistrita Nasaud	BN	Josenii Bargaului	Josenii Bargaului	13.30	–	NA	NA
198	Bistrita Nasaud	BN	Josenii Bargaului	Josenii Bargaului	11.48	–	NA	NA
199	Bistrita Nasaud	BN	Josenii Bargaului	Josenii Bargaului	12.09	–	NA	NA
200	Bistrita Nasaud	BN	Silivasu de Campie	Silivasu de Campie	13.91	–	NA	NA
201	Bistrita Nasaud	BN	Silivasu de Campie	Silivasu de Campie	22.70	–	NA	NA
202	Bistrita Nasaud	BN	Silivasu de Campie	Silivasu de Campie	20.27	–	NA	NA
203	Bistrita Nasaud	BN	Silivasu de Campie	Silivasu de Campie	42.09	+	40	+
204	Bistrita Nasaud	BN	Silivasu de Campie	Silivasu de Campie	22.39	–	NA	NA
205	Bistrita Nasaud	BN	Silivasu de Campie	Silivasu de Campie	14.21	–	NA	NA
206	Bistrita Nasaud	BN	Silivasu de Campie	Silivasu de Campie	13.61	–	NA	NA
207	Bistrita Nasaud	BN	Silivasu de Campie	Silivasu de Campie	13.91	–	NA	NA
208	Bistrita Nasaud	BN	Silivasu de Campie	Silivasu de Campie	23.00	–	NA	NA
209	Bistrita Nasaud	BN	Silivasu de Campie	Silivasu de Campie	16.94	–	NA	NA
210	Bistrita Nasaud	BN		Dumbrava	18.95	–	NA	NA
211	Bistrita Nasaud	BN		Dumbrava	18.25	–	NA	NA
212	Bistrita Nasaud	BN		Dumbrava	17.89	–	NA	NA
213	Bistrita Nasaud	BN		Dumbrava	17.37	–	NA	NA
214	Bistrita Nasaud	BN		Dumbrava	18.60	–	NA	NA
215	Bistrita Nasaud	BN		Dumbrava	18.95	–	NA	NA
216	Bistrita Nasaud	BN		Dumbrava	12.98	–	NA	NA
217	Bistrita Nasaud	BN		Dumbrava	15.44	–	NA	NA
218	Bistrita Nasaud	BN		Dumbrava	16.32	–	NA	NA
219	Bistrita Nasaud	BN		Dumbrava	17.02	–	NA	NA
220	Cluj	CJ	Vad	Vad	71.43	+	0	–
221	Cluj	CJ	Vad	Vad	60.71	+	0	–
222	Cluj	CJ	Vad	Vad	21.43	–	NA	NA
223	Cluj	CJ	Vad	Vad	30.00	+	40	+
224	Cluj	CJ	Vad	Vad	47.14	+	40	+
225	Cluj	CJ	Vad	Calna	49.29	+	10	–
226	Cluj	CJ	Vad	Calna	101.43	+	0	–
227	Cluj	CJ	Vad	Calna	40.00	+	0	–
228	Cluj	CJ	Vad	Calna	55.00	+	0	–

Sample no.	County	County abbreviation	Commune	Locality	ELISA (VIEU/ml)	ELISA result	VNT titer	VNT result
229	Cluj	CJ	Vad	Calna	137.14	+	10	–
230	Cluj	CJ	Floresti	Floresti	10.71	–	NA	NA
231	Cluj	CJ	Floresti	Floresti	12.86	–	NA	NA
232	Cluj	CJ	Floresti	Floresti	52.14	+	0	–
233	Cluj	CJ	Floresti	Floresti	25.00	+	0	–
234	Cluj	CJ	Floresti	Floresti	32.14	+	0	–
235	Cluj	CJ	Floresti	Luna de Sus	49.29	+	10	–
236	Cluj	CJ	Floresti	Luna de Sus	46.43	+	0	–
237	Cluj	CJ	Floresti	Luna de Sus	34.29	+	0	–
238	Cluj	CJ	Floresti	Luna de Sus	33.57	+	80	+
239	Cluj	CJ	Floresti	Luna de Sus	25.71	+	0	–
240	Cluj	CJ	Cluj-Napoca	Cluj-Napoca	118.57	+	20	+
241	Cluj	CJ	Cluj-Napoca	Cluj-Napoca	187.86	+	20	+
242	Cluj	CJ	Cluj-Napoca	Cluj-Napoca	81.43	+	0	–
243	Cluj	CJ	Cluj-Napoca	Cluj-Napoca	37.86	+	10	–
244	Cluj	CJ	Cluj-Napoca	Cluj-Napoca	30.71	+	10	–
245	Cluj	CJ	Cluj-Napoca	Cluj-Napoca	77.14	+	0	–
246	Cluj	CJ	Cluj-Napoca	Cluj-Napoca	66.43	+	0	–
247	Cluj	CJ	Cluj-Napoca	Cluj-Napoca	66.43	+	10	–
248	Cluj	CJ	Cluj-Napoca	Cluj-Napoca	34.29	+	0	–
249	Cluj	CJ	Cluj-Napoca	Cluj-Napoca	110.71	+	10	–
250	Cluj	CJ	Cluj-Napoca	Cluj-Napoca	139.29	+	10	–
251	Cluj	CJ	Cluj-Napoca	Cluj-Napoca	14.29	–	NA	NA
252	Cluj	CJ	Cluj-Napoca	Cluj-Napoca	90.00	+	10	–
253	Cluj	CJ	Cluj-Napoca	Cluj-Napoca	26.43	+	10	–
254	Cluj	CJ	Cluj-Napoca	Cluj-Napoca	25.71	+	20	+
255	Cluj	CJ	Cluj-Napoca	Cluj-Napoca	16.43	–	NA	NA
256	Cluj	CJ	Cluj-Napoca	Cluj-Napoca	40.71	+	10	–
257	Cluj	CJ	Cluj-Napoca	Cluj-Napoca	22.14	–	NA	NA
258	Cluj	CJ	Cluj-Napoca	Cluj-Napoca	14.29	–	NA	NA
259	Cluj	CJ	Cluj-Napoca	Cluj-Napoca	52.14	+	20	+
260	Cluj	CJ	Floresti	Tauti	38.57	+	0	–
261	Cluj	CJ	Floresti	Tauti	22.86	–	NA	NA
262	Cluj	CJ	Floresti	Tauti	57.86	+	0	–
263	Cluj	CJ	Floresti	Tauti	27.14	+	0	–
264	Cluj	CJ	Ciucea	Ciucea	16.43	–	NA	NA
265	Cluj	CJ	Ciucea	Ciucea	85.71	+	10	–
266	Cluj	CJ	Ciucea	Vanatori	117.14	+	10	–
267	Cluj	CJ	Ciucea	Vanatori	26.43	+	10	–
268	Cluj	CJ	Negreni	Negreni	32.86	+	10	–
269	Cluj	CJ	Negreni	Bucea	26.43	+	20	+
270	Cluj	CJ	Ciucea	Ciucea	27.14	+	0	–
271	Cluj	CJ	Ciucea	Ciucea	27.14	+	10	–
272	Cluj	CJ	Ciucea	Ciucea	38.57	+	0	–
273	Cluj	CJ	Ciucea	Ciucea	40.00	+	10	–
274	Cluj	CJ	Ciucea	Ciucea	24.29	–	NA	NA
275	Cluj	CJ	Ciucea	Ciucea	23.57	–	NA	NA
276	Cluj	CJ	Negreni	Negreni	65.00	+	40	+
277	Cluj	CJ	Negreni	Negreni	41.43	+	10	–
278	Cluj	CJ	Ciucea	Ciucea	95.71	+	10	–
279	Cluj	CJ	Ciucea	Ciucea	113.57	+	0	–
280	Cluj	CJ	Gilau	Gilau	34.29	+	10	–
281	Cluj	CJ	Gilau	Gilau	42.86	+	20	+
282	Cluj	CJ	Gilau	Gilau	28.57	+	0	–
283	Cluj	CJ	Gilau	Gilau	22.86	–	NA	NA
284	Cluj	CJ	Gilau	Gilau	50.00	+	10	–
285	Cluj	CJ	Gilau	Gilau	49.29	+	10	–
286	Cluj	CJ	Gilau	Gilau	12.86	–	NA	NA
287	Cluj	CJ	Gilau	Gilau	35.00	+	20	+
288	Cluj	CJ	Gilau	Gilau	32.86	+	10	–
289	Cluj	CJ	Gilau	Gilau	30.71	+	10	–
290	Cluj	CJ	Gilau	Gilau	15.00	–	NA	NA
291	Cluj	CJ	Gilau	Gilau	36.43	+	0	–
292	Cluj	CJ	Gilau	Gilau	25.00	–	NA	NA
293	Cluj	CJ	Gilau	Gilau	22.14	–	NA	NA
294	Cluj	CJ	Gilau	Gilau	15.71	–	NA	NA
295	Cluj	CJ	Gilau	Gilau	11.43	–	NA	NA

Sample no.	County	County abbreviation	Commune	Locality	ELISA (VIEU/ml)	ELISA result	VNT titer	VNT result
296	Cluj	CJ	Gilau	Gilau	30.00	+	0	–
297	Cluj	CJ	Gilau	Gilau	31.43	+	10	–
298	Cluj	CJ	Gilau	Gilau	18.57	–	NA	NA
299	Cluj	CJ	Gilau	Gilau	23.57	–	NA	NA
300	Cluj	CJ	Dabaca	Dabaca	52.14	+	0	–
301	Cluj	CJ	Dabaca	Dabaca	47.14	+	0	–
302	Cluj	CJ	Dabaca	Dabaca	20.00	–	NA	NA
303	Cluj	CJ	Dabaca	Dabaca	27.86	+	0	–
304	Cluj	CJ	Dabaca	Dabaca	20.00	–	NA	NA
305	Cluj	CJ	Dabaca	Dabaca	23.57	–	NA	NA
306	Cluj	CJ	Dabaca	Luna de Jos	43.57	+	0	–
307	Cluj	CJ	Dabaca	Luna de Jos	37.86	+	0	–
308	Cluj	CJ	Dabaca	Luna de Jos	32.14	+	0	–
309	Cluj	CJ	Dabaca	Luna de Jos	14.26	–	NA	NA
310	Cluj	CJ	Dabaca	Luna de Jos	13.91	–	NA	NA
311	Cluj	CJ	Dabaca	Luna de Jos	27.95	+	10	–
312	Cluj	CJ	Dabaca	Paglisa	21.11	–	NA	NA
313	Cluj	CJ	Dabaca	Luna de Jos	18.12	–	NA	NA
314	Cluj	CJ	Dabaca	Paglisa	19.00	–	NA	NA
315	Cluj	CJ	Dabaca	Paglisa	12.86	–	NA	NA
316	Cluj	CJ	Dabaca	Dabaca	14.61	–	NA	NA
317	Cluj	CJ	Dabaca	Dabaca	15.67	–	NA	NA
318	Cluj	CJ	Dabaca	Dabaca	19.00	–	NA	NA
319	Cluj	CJ	Dabaca	Dabaca	14.44	–	NA	NA
320	Mures	MS	Panet	Berghia	12.98	–	NA	NA
321	Mures	MS	Panet	Berghia	12.75	–	NA	NA
322	Mures	MS	Panet	Berghia	37.75	+	20	+
323	Mures	MS	Panet	Berghia	12.30	–	NA	NA
324	Mures	MS	Panet	Berghia	8.43	–	NA	NA
325	Mures	MS	Panet	Berghia	10.93	–	NA	NA
326	Mures	MS	Panet	Berghia	11.39	–	NA	NA
327	Mures	MS	Panet	Hartau	15.48	–	NA	NA
328	Mures	MS	Lunca	Logig	19.57	–	NA	NA
329	Mures	MS	Lunca	Logig	21.61	–	NA	NA
330	Mures	MS	Lunca	Logig	36.84	+	0	–
331	Mures	MS	Lunca	Logig	13.89	–	NA	NA
332	Mures	MS	Lunca	Logig	11.61	–	NA	NA
333	Mures	MS	Galesti	Troita	21.61	–	NA	NA
334	Mures	MS	Galesti	Troita	20.02	–	NA	NA
335	Mures	MS	Galesti	Troita	19.57	–	NA	NA
336	Mures	MS	Galesti	Troita	30.93	+	10	–
337	Mures	MS	Galesti	Troita	25.48	+	10	–
338	Mures	MS	Galesti	Troita	10.93	–	NA	NA
339	Mures	MS	Galesti	Troita	9.11	–	NA	NA
340	Mures	MS	Sanpaul	Sanmarghita	17.07	–	NA	NA
341	Mures	MS	Sanpaul	Sanmarghita	14.57	–	NA	NA
342	Mures	MS	Sanpaul	Sanmarghita	14.11	–	NA	NA
343	Mures	MS	Sanpaul	Sanmarghita	17.98	–	NA	NA
344	Mures	MS	Sanpaul	Valea Izvoarelor	18.20	–	NA	NA
345	Mures	MS	Sanpaul	Valea Izvoarelor	33.66	+	10	–
346	Mures	MS	Sanpaul	Valea Izvoarelor	38.89	+	10	–
347	Mures	MS	Sanpaul	Valea Izvoarelor	19.34	–	NA	NA
348	Mures	MS	Sanpaul	Valea Izvoarelor	14.80	–	NA	NA
349	Mures	MS	Sanpaul	Chirileu	19.57	–	NA	NA
350	Mures	MS	Deda	Bistra Muresului	11.39	–	NA	NA
351	Mures	MS	Deda	Bistra Muresului	20.25	–	NA	NA
352	Mures	MS	Deda	Bistra Muresului	11.39	–	NA	NA
353	Mures	MS	Deda	Bistra Muresului	22.52	–	NA	NA
354	Mures	MS	Deda	Bistra Muresului	11.16	–	NA	NA
355	Mures	MS	Deda	Bistra Muresului	15.93	–	NA	NA
356	Mures	MS	Deda	Bistra Muresului	38.43	+	0	–
357	Mures	MS	Deda	Bistra Muresului	13.89	–	NA	NA
358	Mures	MS	Deda	Bistra Muresului	14.80	–	NA	NA
359	Mures	MS	Deda	Bistra Muresului	16.16	–	NA	NA
360	Mures	MS	Bala	Ercea	28.20	+	0	–
361	Mures	MS	Bala	Ercea	11.16	–	NA	NA
362	Mures	MS	Bala	Ercea	25.93	+	0	–

Sample no.	County	County abbreviation	Commune	Locality	ELISA (VIEU/ml)	ELISA result	VNT titer	VNT result
363	Mures	MS	Bala	Ercea	8.20	–	NA	NA
364	Mures	MS	Bala	Ercea	17.98	–	NA	NA
365	Mures	MS	Bala	Ercea	14.34	–	NA	NA
366	Mures	MS	Bala	Ercea	26.39	+	0	–
367	Mures	MS	Bala	Ercea	15.02	–	NA	NA
368	Mures	MS	Bala	Ercea	10.02	–	NA	NA
369	Mures	MS	Bala	Ercea	16.39	–	NA	NA
370	Mures	MS	Cuci	Cuci	19.57	–	NA	NA
371	Mures	MS	Cuci	Cuci	34.11	+	0	–
372	Mures	MS	Cuci	Cuci	20.02	–	NA	NA
373	Mures	MS	Cuci	Cuci	11.61	–	NA	NA
374	Mures	MS	Cuci	Cuci	30.02	+	20	+
375	Mures	MS	Cuci	Cuci	17.30	–	NA	NA
376	Mures	MS	Cuci	Cuci	23.43	–	NA	NA
377	Mures	MS	Cuci	Cuci	35.70	+	0	–
378	Mures	MS	Cuci	Cuci	19.57	-	NA	NA
379	Mures	MS	Cuci	Cuci	25.70	+	0	–
380	Mures	MS	Iernut	Sfantu Gheorghe	10.70	–	NA	NA
381	Mures	MS	Iernut	Sfantu Gheorghe	23.66	–	NA	NA
382	Mures	MS	Iernut	Sfantu Gheorghe	15.48	–	NA	NA
383	Mures	MS	Iernut	Sfantu Gheorghe	21.16	–	NA	NA
384	Mures	MS	Iernut	Sfantu Gheorghe	15.93	–	NA	NA
385	Mures	MS	Iernut	Sfantu Gheorghe	28.20	+	0	–
386	Mures	MS	Iernut	Sfantu Gheorghe	43.20	+	0	–
387	Mures	MS	Iernut	Sfantu Gheorghe	17.98	–	NA	NA
388	Mures	MS	Solovastru	Solovastru	18.89	–	NA	NA
389	Mures	MS	Solovastru	Solovastru	21.61	–	NA	NA
390	Mures	MS	Solovastru	Solovastru	11.39	–	NA	NA
391	Mures	MS	Solovastru	Solovastru	15.25	–	NA	NA
392	Mures	MS	Solovastru	Solovastru	12.75	–	NA	NA
393	Mures	MS	Solovastru	Solovastru	16.16	–	NA	NA
394	Mures	MS	Solovastru	Solovastru	13.66	–	NA	NA
395	Mures	MS	Solovastru	Solovastru	13.20	–	NA	NA
396	Mures	MS	Solovastru	Jabenita	11.84	–	NA	NA
397	Mures	MS	Solovastru	Jabenita	19.57	–	NA	NA
398	Mures	MS	Solovastru	Jabenita	15.02	–	NA	NA
399	Mures	MS	Solovastru	Jabenita	30.25	–	NA	NA
400	Mures	MS	Rusii Munti	Maioresti	12.30	–	NA	NA
401	Mures	MS	Rusii Munti	Maioresti	8.43	–	NA	NA
402	Mures	MS	Rusii Munti	Maioresti	11.84	–	NA	NA
403	Mures	MS	Rusii Munti	Maioresti	17.30	–	NA	NA
404	Mures	MS	Rusii Munti	Maioresti	19.57	–	NA	NA
405	Mures	MS	Rusii Munti	Maioresti	23.43	–	NA	NA
406	Mures	MS	Rusii Munti	Maioresti	19.57	–	NA	NA
407	Mures	MS	Rusii Munti	Maioresti	14.34	–	NA	NA
408	Mures	MS	Rusii Munti	Maioresti	17.98	–	NA	NA
409	Mures	MS	Rusii Munti	Maioresti	12.07	–	NA	NA
410	Mures	MS	Batos	Uila	18.30	–	NA	NA
411	Mures	MS	Batos	Uila	15.14	–	NA	NA
412	Mures	MS	Batos	Uila	23.21	–	NA	NA
413	Mures	MS	Batos	Uila	15.14	–	NA	NA
414	Mures	MS	Batos	Uila	22.68	–	NA	NA
415	Mures	MS	Batos	Uila	13.56	–	NA	NA
416	Mures	MS	Batos	Uila	12.68	–	NA	NA
417	Mures	MS	Batos	Uila	13.56	–	NA	NA
418	Mures	MS	Batos	Uila	13.39	–	NA	NA
419	Mures	MS	Batos	Uila	17.42	–	NA	NA
420	Salaj	SJ	Garbou	Garbou	22.78	–	NA	NA
421	Salaj	SJ	Garbou	Garbou	25.08	+	10	–
422	Salaj	SJ	Garbou	Garbou	28.78	+	20	+
423	Salaj	SJ	Garbou	Garbou	21.08	–	NA	NA
424	Salaj	SJ	Garbou	Garbou	23.24	–	NA	NA
425	Salaj	SJ	Garbou	Garbou	26.78	+	20	+
426	Salaj	SJ	Garbou	Garbou	27.85	+	10	–
427	Salaj	SJ	Garbou	Garbou	19.39	–	NA	NA
428	Salaj	SJ	Garbou	Garbou	27.55	+	10	–
429	Salaj	SJ	Garbou	Garbou	24.01	–	NA	NA

Sample no.	County	County abbreviation	Commune	Locality	ELISA (VIEU/ml)	ELISA result	VNT titer	VNT result
430	Salaj	SJ	Hida	Hida	22.16	–	NA	NA
431	Salaj	SJ	Hida	Hida	23.55	–	NA	NA
432	Salaj	SJ	Hida	Hida	21.24	–	NA	NA
433	Salaj	SJ	Hida	Hida	24.93	–	NA	NA
434	Salaj	SJ	Hida	Hida	25.39	+	10	–
435	Salaj	SJ	Hida	Hida	21.55	-	NA	NA
436	Salaj	SJ	Hida	Hida	25.24	+	40	+
437	Salaj	SJ	Hida	Hida	20.78	–	NA	NA
438	Salaj	SJ	Hida	Hida	23.85	–	NA	NA
439	Salaj	SJ	Hida	Hida	23.39	–	NA	NA
440	Salaj	SJ	Balan	Balan	20.78	–	NA	NA
441	Salaj	SJ	Balan	Balan	21.70	–	NA	NA
442	Salaj	SJ	Balan	Balan	24.78	–	NA	NA
443	Salaj	SJ	Balan	Balan	23.70	–	NA	NA
444	Salaj	SJ	Balan	Balan	25.24	+	40	+
445	Salaj	SJ	Balan	Balan	18.62	–	NA	NA
446	Salaj	SJ	Balan	Balan	20.47	–	NA	NA
447	Salaj	SJ	Balan	Balan	21.70	–	NA	NA
448	Salaj	SJ	Balan	Balan	19.55	–	NA	NA
449	Salaj	SJ	Balan	Balan	22.62	–	NA	NA
450	Salaj	SJ	Dragu	Dragu	22.78	–	NA	NA
451	Salaj	SJ	Dragu	Dragu	19.55	–	NA	NA
452	Salaj	SJ	Dragu	Dragu	24.01	–	NA	NA
453	Salaj	SJ	Dragu	Dragu	31.85	+	10	–
454	Salaj	SJ	Dragu	Dragu	21.70	–	NA	NA
455	Salaj	SJ	Dragu	Dragu	19.85	–	NA	NA
456	Salaj	SJ	Dragu	Dragu	19.85	–	NA	NA
457	Salaj	SJ	Dragu	Dragu	20.93	–	NA	NA
458	Salaj	SJ	Dragu	Dragu	21.39	–	NA	NA
459	Salaj	SJ	Dragu	Dragu	26.32	+	40	+
460	Salaj	SJ	Letca	Letca	28.16	+	20	+
461	Salaj	SJ	Letca	Letca	21.55	–	NA	NA
462	Salaj	SJ	Letca	Letca	40.78	+	20	+
463	Salaj	SJ	Letca	Letca	18.47	–	NA	NA
464	Salaj	SJ	Letca	Letca	22.32	–	NA	NA
465	Salaj	SJ	Letca	Letca	19.39	–	NA	NA
466	Salaj	SJ	Letca	Letca	17.70	–	NA	NA
467	Salaj	SJ	Letca	Letca	18.47	–	NA	NA
468	Salaj	SJ	Letca	Letca	37.55	+	40	+
469	Salaj	SJ	Letca	Letca	31.08	+	20	+
470	Salaj	SJ	Dobrin	Dobrin	29.08	+	20	+
471	Salaj	SJ	Dobrin	Dobrin	24.16	–	NA	NA
472	Salaj	SJ	Dobrin	Dobrin	18.16	–	NA	NA
473	Salaj	SJ	Dobrin	Dobrin	18.62	–	NA	NA
474	Salaj	SJ	Dobrin	Dobrin	28.01	+	40	+
475	Salaj	SJ	Dobrin	Dobrin	24.93	–	NA	NA
476	Salaj	SJ	Dobrin	Dobrin	24.01	–	NA	NA
477	Salaj	SJ	Dobrin	Dobrin	26.93	+	40	+
478	Salaj	SJ	Dobrin	Dobrin	31.08	+	20	+
479	Salaj	SJ	Dobrin	Dobrin	32.47	+	10	–
480	Salaj	SJ	Cuzaplac	Cuzaplac	21.85	–	NA	NA
481	Salaj	SJ	Cuzaplac	Cuzaplac	20.01	–	NA	NA
482	Salaj	SJ	Cuzaplac	Cuzaplac	44.01	+	20	+
483	Salaj	SJ	Cuzaplac	Cuzaplac	29.55	+	20	+
484	Salaj	SJ	Cuzaplac	Cuzaplac	58.93	+	10	–
485	Salaj	SJ	Cuzaplac	Cuzaplac	26.01	+	80	+
486	Salaj	SJ	Cuzaplac	Cuzaplac	18.93	–	NA	NA
487	Salaj	SJ	Cuzaplac	Cuzaplac	21.08	–	NA	NA
488	Salaj	SJ	Cuzaplac	Cuzaplac	28.01	+	40	+
489	Salaj	SJ	Cuzaplac	Cuzaplac	21.39	–	NA	NA
490	Salaj	SJ	Mesesenii de Jos	Mesesenii de Jos	23.55	–	NA	NA
491	Salaj	SJ	Mesesenii de Jos	Mesesenii de Jos	21.24	–	NA	NA
492	Salaj	SJ	Mesesenii de Jos	Mesesenii de Jos	19.39	–	NA	NA

Sample no.	County	County abbreviation	Commune	Locality	ELISA (VIEU/ml)	ELISA result	VNT titer	VNT result
493	Salaj	SJ	Mesesenii de Jos	Mesesenii de Jos	25.55	+	0	–
494	Salaj	SJ	Mesesenii de Jos	Mesesenii de Jos	21.08	–	NA	NA
495	Salaj	SJ	Mesesenii de Jos	Mesesenii de Jos	23.70	–	NA	NA
496	Salaj	SJ	Mesesenii de Jos	Mesesenii de Jos	22.78	–	NA	NA
497	Salaj	SJ	Mesesenii de Jos	Mesesenii de Jos	25.24	+	80	+
498	Salaj	SJ	Mesesenii de Jos	Mesesenii de Jos	24.16	-	NA	NA
499	Salaj	SJ	Mesesenii de Jos	Mesesenii de Jos	45.24	+	10	–
500	Salaj	SJ	Fildu de Jos	Fildu de Jos	21.24	–	NA	NA
501	Salaj	SJ	Fildu de Jos	Fildu de Jos	19.08	–	NA	NA
502	Salaj	SJ	Fildu de Jos	Fildu de Jos	23.70	–	NA	NA
503	Salaj	SJ	Fildu de Jos	Fildu de Jos	21.70	–	NA	NA
504	Salaj	SJ	Fildu de Jos	Fildu de Jos	21.24	–	NA	NA
505	Salaj	SJ	Fildu de Jos	Fildu de Jos	23.55	–	NA	NA
506	Salaj	SJ	Fildu de Jos	Fildu de Jos	21.39	–	NA	NA
507	Salaj	SJ	Fildu de Jos	Fildu de Jos	21.39	–	NA	NA
508	Salaj	SJ	Fildu de Jos	Fildu de Jos	20.62	–	NA	NA
509	Salaj	SJ	Fildu de Jos	Fildu de Jos	24.93	–	NA	NA
510	Salaj	SJ	Galgau	Galgau	32.00	+	0	–
511	Salaj	SJ	Galgau	Galgau	26.08	+	20	+
512	Salaj	SJ	Galgau	Galgau	21.80	–	NA	NA
513	Salaj	SJ	Galgau	Galgau	33.63	+	0	–
514	Salaj	SJ	Galgau	Galgau	22.61	–	NA	NA
515	Salaj	SJ	Galgau	Galgau	25.88	+	0	–
516	Salaj	SJ	Galgau	Galgau	18.94	–	NA	NA
517	Salaj	SJ	Galgau	Galgau	21.39	–	NA	NA
518	Salaj	SJ	Galgau	Galgau	34.24	+	20	+
519	Salaj	SJ	Galgau	Galgau	16.90	–	NA	NA

*NA, not applicable; ELISA, enzyme-linked immunosorbent assay; VNT, virus neutralization test.
